# Cytotoxic Effects on Gingival Mesenchymal Stromal Cells and Root Surface Modifications Induced by Some Local Antimicrobial Products Used in Periodontitis Treatment

**DOI:** 10.3390/ma14175049

**Published:** 2021-09-03

**Authors:** Irina Lupșe, Emoke Pall, Lucian Barbu Tudoran, Adriana Elena Bulboacă, Andreea Ciurea, Iulia Cristina Micu, Alexandra Roman, Ada Gabriela Delean, Alexandrina Muntean, Andrada Soancă

**Affiliations:** 1 Department of Paediatric Dentistry, Faculty of Dental Medicine, Iuliu Hațieganu University of Medicine and Pharmacy, Avram Iancu St., No. 31, 400083 Cluj-Napoca, Romania; irinalupse@yahoo.com (I.L.); ortoanda@yahoo.com (A.M.); 2 Department of Infectious Disease, Faculty of Veterinary Medicine, University of Agricultural Sciences and Veterinary Medicine, 3-5 Mănăștur St., 400372 Cluj-Napoca, Romania; pallemoke@gmail.com; 3 Department of Molecular Biology and Biotechnologies, Faculty of Biology and Geology, Babeş-Bolyai University, Clinicilor St., No. 5-7, 400006 Cluj-Napoca, Romania; lucianbarbu@yahoo.com; 4 Electron Microscopy Integrated Laboratory (LIME), National Institute for Research and Development of Isotopic and Molecular Technologies, INCDTIM, 67-103 Donath St., 400293 Cluj-Napoca, Romania; 5 Department of Pathophysiology, Iuliu Hațieganu University of Medicine and Pharmacy, 4-6 Victor Babeș St., 400012 Cluj-Napoca, Romania; adriana_bulboaca@yahoo.com; 6 Department of Periodontology, Faculty of Dental Medicine, Iuliu Haţieganu University of Medicine and Pharmacy, Victor Babeş St., No. 15, 400012 Cluj-Napoca, Romania; andreea_candea@yahoo.com (A.C.); i.cristina.micu@gmail.com (I.C.M.); andrapopovici@gmail.com (A.S.); 7 Department of Odontology and Endodontics, Faculty of Dental Medicine, Iuliu Haţieganu University of Medicine and Pharmacy, 400012 Cluj-Napoca, Romania; adadelean@yahoo.com

**Keywords:** stem cell, adhesion, dental disinfectant, smear layer, tooth root

## Abstract

(1) Background: this study aims to test the cytotoxicity of three antimicrobial products used in periodontitis treatment on gingival mesenchymal stem cells (gMSCs) and their influence on root surfaces and gMSC adhesion. We tested the null hypothesis that the effects of the antimicrobials did not differ. (2) Methods: the commercial products based on sulphonic/sulphuric acids, sodium hypochlorite and silver nanoparticles, in five different concentrations, were added to culture medium for growing gMSCs. Cell proliferation capacity was tested using the Cell Counting Kit-8 (CCK8) and their viability was determined by succinate dehydrogenase activity (MTT) assay. Scanning electron microscopy evaluated the adhesion of gMSCs on root samples treated mechanically and with commercial products. (3) Results: the products induced a dose-dependent cytotoxicity in terms of reduced proliferation and viability of gMSCs, as well as cell shape modifications. Significant differences in CCK8 values between the different commercial products were observed. Based on proliferation tests, the null hypothesis was rejected. When MTT values of the three products were compared with each other, no significant differences were observed for any of the five concentrations (*p* = 0.065, *p* = 0.067, *p* = 0.172, *p* = 0.256, *p* = 0.060). (4) Conclusions: the three antimicrobials had a certain degree of cytotoxicity on gMSCs. gMSCs repopulated treated root surfaces.

## 1. Introduction

Periodontitis is an infectious disease which leads to the inflammatory destruction of the periodontal ligament and alveolar bone [1]. Severe forms of periodontitis could induce tooth loss as well as important systemic consequences [2]. Treatment of periodontitis firstly aims to prevent further periodontal loss through severe reduction of local bacterial load [3] by professional subgingival instrumentation and patient-related approaches. By eliminating the infectious aetiology from the subgingival areas, subgingival instrumentation is considered the gold standard of periodontal therapy in all forms of periodontitis [4] and its clinical efficacy is well documented by previous or more recent systematic reviews [5,6,7,8]. Deep periodontal pockets or local anatomical risk factors such as furcation involvement or root concavities limit the access of professional subgingival instrumentation, thus, resulting in the persistence of the periodontal infection [9]. The use of locally delivered adjunctive products during subgingival instrumentation in periodontitis patients could improve clinical outcomes [5,7] as it has been shown that this therapy provides an antimicrobial effect [10] and facilitates the removal of subgingival deposits, dead tissue debris, and inflammatory exudates [11,12]. Nonetheless, the possible deleterious effects of the subgingival application of adjunctive antimicrobials on periodontal constituents also needs to be considered [13,14].

Periodontal tissues contain mesenchymal stromal cells (MSCs) [15] capable of restoring structure and function, but the intrinsic regenerative capacity of the periodontium is limited [16]. Periodontal MSCs communicate with their immediate surroundings and adjacent cells yielding cell-based responses which are deemed to be therapeutically favourable in terms of tissue reconstruction [15]. However, minimal changes in the microenvironment affect the behaviour of MSCs and their regenerative potential [17]. Thus, the protection of local cells against potential harmful effects of some periodontal therapies appears to be a rational target for treating periodontitis.

Some commercial products based on chlorhexidine, doxycycline hyclate or minocycline gel [7], metronidazole gel [5], sodium hypochlorite [18], povidone iodine [19], silver nanospheres [20] or sulphonic acids [21,22,23] are currently being used in clinical practice as local adjunctive therapies to subgingival instrumentation mostly due to their antimicrobial effect. However, some drawbacks have been related to their use in terms of clinical performance [20] and biological effects [24,25,26,27,28,29]. Chlorhexidine showed a cytotoxic effect on human stem cells [24], alveolar bone cells [14], gingival epithelial cells [13] in a concentration—and time—dependent manner [24]. Chlorhexidine had a lower cytotoxicity compared to sodium hypochlorite [25], but induced no cellular survival at the minimal bactericidal concentration [26]. Different concentrations of sodium hypochlorite exhibited deleterious effects on stem cells of the apical papilla [27] and human bone marrow MSCs [28] in a direct relationship with higher concentrations and increased exposure time [28]. Sodium hypochlorite is included in endodontic as well as in periodontal products [29].

Povidone-iodine is an antimicrobial substance that negatively influences cell survival and proliferation of human osteoblast, fibroblast and myoblast cells [30]. Silver nanoparticles are promising antimicrobial and anti-inflammatory systems that display antitumor activity and sustained drug-carrier potential [31]. Additionally, silver nanoparticles induce a cytotoxic effect on human periodontal fibroblasts [32] and MSCs [33].

To date, the clinical relevance of the utility of adjunctive local antimicrobials in association with subgingival instrumentation in the treatment of periodontitis is still uncertain [4,20] and no consensus regarding the most effective substances or recommended concentration has been available. Some local antimicrobial products based on sulfonic/sulphuric acids, silver nanoparticles or sodium hypochlorite are available for the treatment of periodontitis, but there are few studies to support their biocompatibility with periodontal tissues. Conducting further investigations could bring positive evidence in this regard and arguments to increase their clinical utility preferentially over other products.

A dual antimicrobial desiccant product containing a blend of sulphonic/sulphuric acids has been recently proposed as adjunctive therapy to subgingival instrumentation [21,22,23]. These acids have a strong affinity to bind to the water retained by the biofilm matrix and they have been demonstrated to cause molecular denaturation and tissue coagulation of the outermost layer of periodontal tissue. The irreversible rapid desiccation mechanism would allow a quick detachment of the subgingival calculus and a destruction of the biofilm [12]. However, desiccant products have not been well studied and their mechanism of action is not completely understood [20]. Information on the cytotoxicity of sulphonic/sulphuric acids-based local products on oral cells is very limited. Moreover, some toxicological aspects associated with the use of silver nanoparticle-based systems should further be elucidated [31] and there are very few data on their effect on oral cells. Additionally, information on the impact of the above-mentioned products on root surfaces is scarce.

The present study addresses the underlying inconsistencies in the scientific literature related to the microscopical impact of local adjunctive antimicrobials associated with subgingival instrumentation. It, therefore, aims to investigate the effect exerted by some less studied commercial products based on sulphonic/sulphuric acids, sodium hypochlorite and silver nanoparticles, respectively, on the proliferation and viability of MSCs isolated from gingival tissues (gMSCs). For the purpose of this study, the following null hypothesis was tested: the biocompatibility of the tested commercial products did not differ with different product concentrations. The study also used SEM analysis to evaluate the influence of the root surface modifications induced by these products on root surfaces and gMSC adhesion.

## 2. Materials and Methods

### 2.1. Study Design

The protocol used in this study was approved by the Ethical Board of the Iuliu Haţieganu University (No. 472/19 December 2018). Prior to tissue sample and tooth collection, all patients were informed of the study protocol and of all procedural details of our research, and they were asked to give signed informed consent. The experiments included in this present study meet the principles outlined in the Declaration of Helsinki on experimentation involving human subjects and were conducted in accordance with EU and national laws.

In order to observe the influence of antimicrobial products on gMSCs, experimental culture media were prepared by supplementing the standard culture medium with different concentrations of three commercially antimicrobial products mostly used as adjunctive materials to subgingival instrumentation in periodontitis treatment. The proliferation capacity of gMSCs was tested using the Cell Counting Kit-8 (CCK8) method, after 48 h and 5 days of culture in experimental growth media. Moreover, after 24 h of gMSC culture in experimental media, MTT assay was performed to evaluate cell survival as an expression of cytotoxicity induced by the experimental substances. For the control group, gMSCs cultivated in standard culture medium were used.

In a second phase, the study employed SEM observation to evaluate the modifications of dental root surfaces induced by mechanical subgingival instrumentation associated with each of the commercial products. SEM evaluation also observed the adhesion of gMSCs on the treated surfaces.

All experiments were performed in triplicate.

### 2.2. Gingival Mesenchymal Stromal Cell (gMSC) Isolation and Characterization

The gMSCs were obtained from a 34-year-old healthy female smoker, from normal gingival tissues resulted during a mucogingival plastic surgery. gMSC were already fully characterized (results elsewhere—Stanomir et al., unpublished data) following the achievement of the standard minimal criteria suggested by the International Society for Cellular Therapy [34]: specific surface antigen make-up and trilineage differentiation potential. For the present study, cells after four passages were used.

### 2.3. Preparation of Experimental Culture Media

Three commercially antimicrobial products already in use for periodontal subgingival instrumentation were tested: HybenX (Epien Medical, St. Paul, MN, USA) (HI), Perisolv (RLS Global, Gothenburg, Sweden) (PS), Perioflush (Dental Life Sciences, Wigan, UK) (PF) (Table 1).

The experimental solutions were prepared in five different concentrations (50%, 20%, 10%, 5%, 2%) for each commercial product, by diluting them with the corresponding volume of sterile phosphate saline (PBS) (Lonza, Basel, Switzerland). To evaluate the direct effect of the antimicrobial products on gMSCs, each concentration of the experimental solutions was supplemented in standard culture medium (Dulbecco’s Modified Eagle’s Medium/Nutrient F-12 Ham/DMEM/F12—Sigma-Aldrich, St. Louis, MO, USA, plus 10% FCS—EuroClone, Pero, MI, Italy, plus 1% antibiotic/antimycotic—Sigma-Aldrich) at a ratio of 1:10.

### 2.4. Cell Proliferation (CCK-8)

A Cell Counting Kit-8 (Sigma Aldrich) was used for this assay, after 48 h and 5 days of growing gMSCs in experimental media. To evaluate the proliferation of gMSCs, 500 μL of cell suspension (3 × 10^4^ cells) was cultivated in three 24-well plates (Thermo Fisher Scientific, Waltham, MA, USA) with 50 μL of each concentration of the experimental solutions (HI, PS, PF at 50%, 20%, 10%, 5%, 2%). After 48 h and 5 days, a total volume of 50 μL of the CCK-8 solution was added to each well. The plate was then incubated for 4 h. Before evaluation, the cells were rinsed with PBS (Lonza). The optical densities of each well were read at 450 nm with a Synergy 2 microplate reader (BioTek, Winooski, VT, USA).

### 2.5. Cell Survival by MTT Assay

Cells were allowed to adhere for 24 h to 96-well plates (1 × 10^4^ cells) in standard culture medium. The cell media was then replaced with 200 μL fresh media supplemented with 20 μL of various dilutions of each commercial product (HybenX, Perisolv, Perioflush at 50%, 20%, 10%, 5%, 2%). Standard medium served as a control. Following exposure to the solutions for 24 h, 10 μL of 5 mg/mL MTT solution (3-(4,5-dimethylthiazol-2-yl)-2,5-diphenyltetrazolium bromide—Sigma–Aldrich) in PBS (Lonza) were added in each well. MTT is a tetrazolium salt that is converted by cell mitochondrial reductases of viable cells into a dark blue compound-formazan. After incubation for 4 h at 37 °C in dark, the MTT solution was removed from each well and 100 μL DMSO dye (dimethylsulfoxide) (Fluka, Buchs, Switzerland) was added. Before evaluation, the cells were rinsed with PBS (Lonza). The optical density of the colour reaction at 450 nm was determined with a Synergy 2 microplate reader (BioTek).

### 2.6. Cell Culture on Treated Root Samples

**Figure 1:**
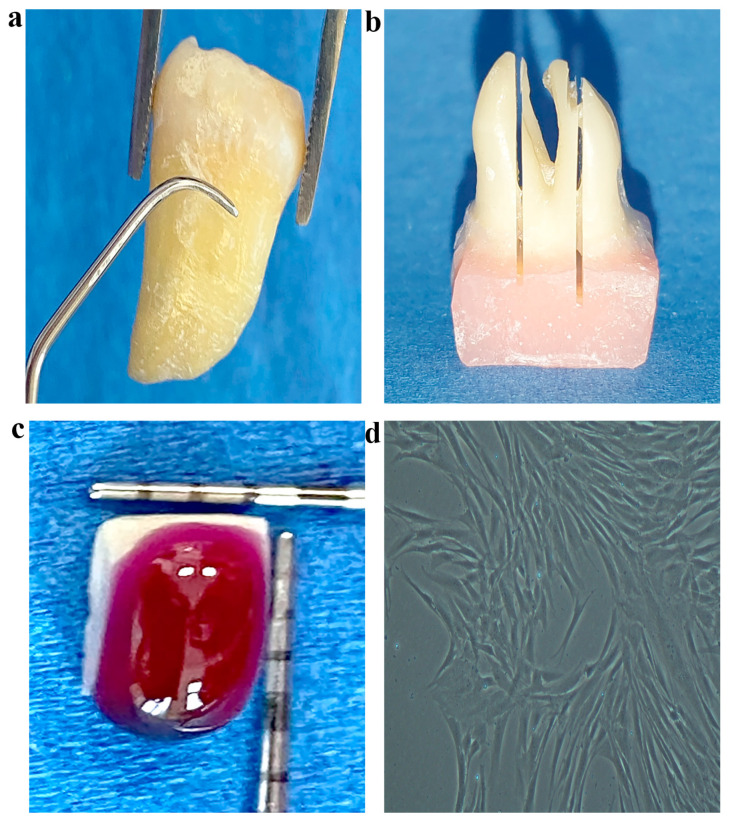
Sample preparation for SEM analysis. (**a**) Root scaling; (**b**) longitudinally sectioned roots; (**c**) HI on a standard root sample; (**d**) MSCs prepared to be harvested at 80% confluence.

Eight human inferior molars were used for the present experiment. Tooth collection was performed following relevant guidelines and regulations. Teeth were extracted for orthodontic or periodontal reasons, which were not related to the present study. The extracted teeth were stored in 4% chloramine-T after the removal of soft tissue debris and they were used within one month after extraction. SRP was performed for all roots with a Gracey curette no. 7/8 (Hu-Friedy, Chicago, IL, USA) by applying five long movements in a cervical-apical direction on each root area (Figure 1a). One operator (IL) previously trained performed all instrumentations. The teeth were rinsed in saline solution. The crowns were embedded in autopolymerizing acrylic resin (Duracryl^®^ Plus, SpofaDental, Czech Republic) using a silicon mounting template (Zetaplus & Indurent gel, Zhermack, Badia Polesine, Italy) leaving the roots exposed (Figure S1a). Each root was sectioned in a buccal-oral and apical-coronal direction using a low-speed diamond saw (Isomet, Buehler Ltd., Illinois, IL, USA), resulting in two external sections of about 2 mm in thickness and one central part (Figure 1b). In order to evaluate the changes of root surface and the adhesion of gMSCs, root samples were prepared from the external root sections (Figure S1b) using diamond-coated burs (Komet 6837.314.012; Komet, Lemgo, Germany) mounted on a high-speed hand-piece with air and water cooling. A total of 24 root samples (Figure S1c) of 4 × 3 × 2 mm each (Figure 1c) were prepared from the cervical thirds of the root sections, 1 mm apically to the cement-enamel junction [38]. All samples were sterilized using ethylene oxide gas for 2 h at 56 °C followed by degassing for 12 h. Just before placement in culture media, samples were treated with commercial products according to manufacturer’s instructions (30 s for HI and PS and 2 min for PF) and then thoroughly rinsed with PBS solution (Lonza).

Three samples were treated with each commercial product and three other samples that had not been chemically treated were used as controls. The 12 root samples were placed in a culture medium without cells in order to observe surface microscopical changes induced by the treatment. Other 12 identically treated samples were placed in 24-well Petri dishes to observe gMSC adhesion through SEM. Then, 10^3^ cells obtained from culture at 80% confluence (Figure 1d) were plated on top of these samples. One day after seeding, the root samples were fixed in 2% glutaraldehyde in a 0.1 M PBS (Lonza) (pH = 7.4) overnight at 4 °C for SEM analysis.

### 2.7. Statistical Analysis

Statistical analysis was performed using the MedCalc^®^ Statistical Software version 19.7.1 (MedCalc Software Ltd., Ostend, Belgium; https://www.medcalc.org, accessed on 28 February 2021). Data were expressed as mean and standard deviation (±SD) (normal distribution). Comparison between groups was performed using Analysis of Variance (ANOVA). Differences between measurements, taking into consideration different groups, were verified using two-way repeated measures ANOVA. A *p* value lower than 0.05 was considered statistically significant.

## 3. Results

### 3.1. CCK Analysis

**Figure 2:**
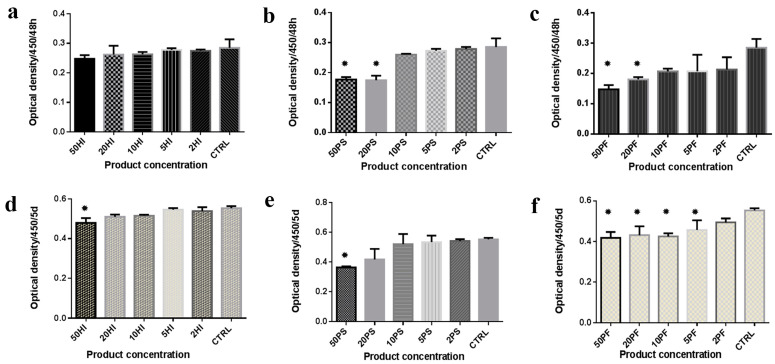
Proliferation after 48 h and 5 days of culture in different media. The whiskers correspond to the standard deviation. Results after 48 h for experimental media containing: (**a**) HI; (**b**) PS; (**c**) PF; results after 5 days for experimental media containing (**d**) HI; (**e**) PS; (**f**) PF; HI = HybenX; PS = Perisolv; PF = Perioflush; CTRL = Control. * = statistically different vs. controls.

**Figure 3:**
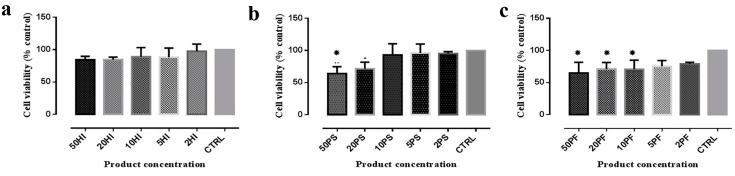
Graphical representation of MTT test. The whiskers correspond to the standard deviation. Results for experimental media containing: (**a**) HI; (**b**) PS; (**c**) PF; HI = HybenX; PS = Perisolv; PF = Perioflush; CTRL = Control. * = statistically different vs. controls.

The cells were grown in experimental culture media containing different concentrations of commercial products and their proliferation and viability capacities were tested using the Cell Counting Kit-8 (CCK8) method and MTT assay, respectively. The results are provided in Figure 2 and Figure 3 as well as in Table S1.

After 48 h, the optical density values indicating cell proliferation after exposure to various concentrations of HI were not significantly different (*p* = 0.226) (Figure 2a). After 5 days, the concentration of 50% HI significantly inhibited cell proliferation in comparison with 5% HI (*p* = 0.001), 2% HI (*p* = 0.003) and control medium (*p* = 0.001) (Figure 2d).

After 48 h of exposure to different concentrations of PS, cells grown in 50% PS proliferated less than cells grown in all the other media except for 20% PS (all values of *p* < 0.001). The same trend was remarked for 20% PS concentration (excepting for that related to 50% PS, all values of *p* < 0.001) (Figure 2b). After 5 days of PS exposure, cells proliferated significantly differently: the 50% PS containing medium significantly inhibited cells in comparison with all the other media except for 20% PS (*p* = 0.009, *p* = 0.005, *p* = 0.004, *p* = 0.002, respectively) (Figure 2e).

For PF exposure, the optical density evaluation after 48 h indicates proliferation values significantly lower for cells grown in 50% PF and 20% PF in comparison with the control medium (*p* = 0.002, *p* = 0.014, respectively) (Figure 2c). After 5 days of exposure to different concentrations of PF, significant differences in optical density values were recorded: except for the lowest concentration (2%), all the other PF concentrations induced significantly less cell proliferation in comparison with the control medium (*p* = 0.002, *p* = 0.004, *p* = 0.003, *p* = 0.024, respectively) (Figure 2f).

When the three products were compared with each other for equivalent concentrations, some significant differences of CCK values were found for both moments of measurement. Thus, inter-group comparisons for the 50% concentrations provided significant differences in proliferation values for both 48 h-CCK8 and 5 days-CCK8. For 48 h-CCK8 significant differences were found between 50% HI vs. 50% PS (*p* = 0.001) and 50% HI vs. 50% PF (*p* < 0.001) and for 5 days-CCK8 between 50% HI vs. 50% PS (*p* = 0.002). For the 20% concentrations, significant differences were calculated between 48 h-CCK8 values: 20% HI vs. 20% PS (*p* = 0. 004) and 20% HI vs. 20% PF (*p* = 0.006). For 48 h-CCK, when 10% concentrations were compared, significant differences were found between 10% HI vs. 10% PF (*p* < 0.01) and 10% PS vs. 10% PF (*p* < 0.01). A significant difference was also found between 10% PS vs. 10% PF for 5 days-CCK8 (*p* = 0.058). Additionally, significant differences were found for 2% concentrations for 48 h-CCK8 between 2% HI vs. 2% PF (*p* = 0.045) and 2% PS vs. 2% PF (*p* = 0.036) and for 5 days-CCK8 between 2% PS vs. 2% PF (*p* = 0.036).

### 3.2. MTT Analysis

At 24 h, the optical density values indicating cell viability after HI exposure showed no significant differences when the different concentrations were compared: (*p* = 0.390) (Figure 3a). The intragroup comparisons for PS exposure reveal that vitality values were significantly lower for cells grown in 50% PS than for cells grown in 5% or 2% PS containing media as well as in the control medium (*p* = 0.025. *p* = 0.032, *p* = 0.012, respectively) (Figure 3b).

For PF exposure, (Figure 3c), cells grown in 50%, 20%, and 10% PF had a decreased viability compared to the cells grown in the control medium (*p* = 0.003, *p* = 0.015, *p* = 0.013, respectively).

When MTT values of equivalent concentrations of the three products were compared, no significant differences were observed for any of the five concentrations (*p* = 0.065, *p* = 0.067, *p* = 0.172, *p* = 0.256, *p* = 0.060).

### 3.3. SEM Observations

**Figure 4:**
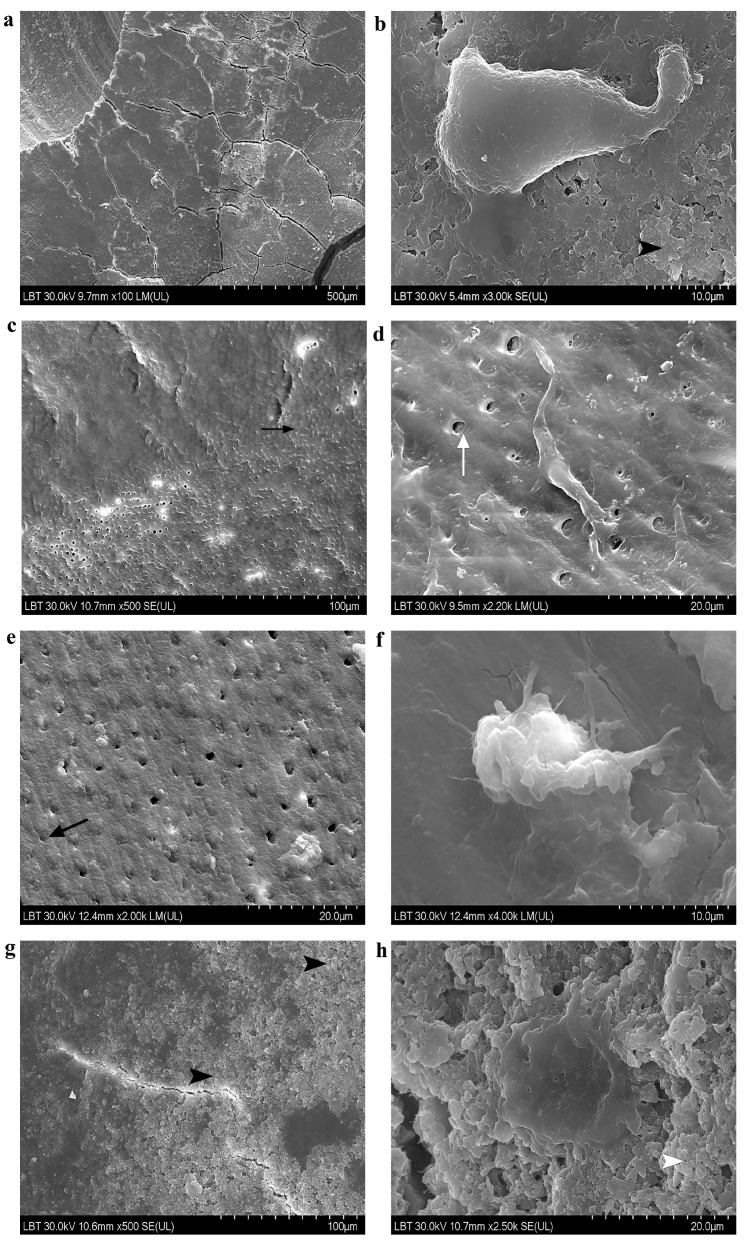
SEM observed samples. (**a**) Continuous smear layer on a instrumented root sample (100×); (**b**) elongate cell, well-attached through pseudopods on a rough smear-layer covered sample after subgingival instrumentation (3000×); (**c**) transversally sectioned dentin tubuli partly obstructed by a continuous smooth smear layer (upper part) on a HI-treated and instrumented sample (500×); (**d**) fusiform well-attached cell through long lamellipodia and dentin tubuli of 3-4 µm on a HI-treated and instrumented sample (2200×); (**e**) relative smooth smear layer surface partially obstructing dentin tubuli of a PS-treated and instrumented sample (2000×); (**f**) attached cell through filopodia on a relatively smooth PS-treated and instrumented sample (4000×); (**g**) rough surface due to important deposits of a PF-treated and instrumented sample (500×); (**h**) beautiful flattened adherent cell through short lamellipodia on a very rough PF-treated and instrumented sample (2500×)**.** HI = HybenX; PS = Perisolv; PF = Perioflush. Black arrowhead = rough smear-layer; white arrowhead = very rough smear layer; black arrow = partly obstructed dentin tubuli; white arrow = open dentin tubuli.

SEM observations evaluated the modifications of the root samples after mechanical instrumentation plus commercial product applications and the adhesion of gMSCs on the treated samples. Mechanically instrumented samples are generally covered by a continuous smear layer rendering an overall smooth appearance (Figure 4a). HI-treated and instrumented samples show areas of continuous smooth compact smear layer alternating with areas of partially opened dentin tubuli (Figure 4c,d). More randomly open tubuli are distributed on PS-treated and instrumented surfaces (Figure 4e). PF-treated and instrumented samples display an abundant deposit that renders a rough appearance of the surfaces (Figure 4g,h). Many samples are furrowed by cracks—a normal consequence of the methodology dehydration process (Figure 4a).

On cell-treated samples, rare cells colonize the root surfaces. Although extremely elongated in 70–80% confluence on culture media, cells acquired a rounder form when cultured on root fragments treated with PS and PF plus mechanical instrumentation (Figure 4f,h). gMSCs cultured on HI-treated and instrumented samples display a more elongated shape (Figure 4d). Cells were well attached through lamellipodia and filopodia (Figure 4b,d,f,h).

## 4. Discussion

The present study examined the influence of three commercial antimicrobial products used as local adjunctive of mechanical subgingival instrumentation on the proliferation and survival of MSCs isolated from human gingiva through CCK8 test and MTT assay, respectively. Additionally, the study appreciated the morphological changes of root surfaces following mechanical instrumentation and antimicrobial applications in relation to gMSC adhesion. Cell adhesion and proliferation are important steps in periodontal healing after various therapies [38]. The nature of root surfaces provided by mechanical subgingival instrumentation and local antimicrobial applications used in periodontitis treatment could influence local reparatory phenomena [39]. These phenomena could also be impacted by the interactions of periodontal progenitor pools with locally delivered materials.

This study observed an inversely proportional trend between the concentrations of antimicrobial products in the culture media and the proliferation and viability of gMSCs. HI seemed relatively better tolerated than the other products, since only 50% HI provided a significant inhibitory proliferative effect after five days (CCK8 test) in comparison with controls. For PS both 50% and 20% concentrations induced a certain cytotoxicity in comparison with lower concentrations or controls, as revealed by MTT and CCK8 assays and five days-CCK8 assays, respectively. The PF in concentrations of 50%, 20% and 10% witnessed a significant cytotoxicity in terms of cell viability (MTT assay) and 5 days-proliferation (CCK8 test).

The present results reject the null hypothesis because there were significant differences in the toxicity of the products on gMSC, when corresponding concentrations were compared. However, the results should be regarded with caution due to the reduced sample size, which is considered to be a limitation of this study. Further investigations on an extended number of samples should be conducted.

To our knowledge, this present study is the first to provide data on HI toxic effects on gMSCs. Apart from the positive results furnished by standard cytotoxicity tests [36], it appears that no other published information concerning HI direct influence on oral cells is available. However, it was observed that when used for direct pulp capping in dogs, this product was more favourable to cell vitality and new dentin formation than conventional calcium hydroxide products [40]. HI is a novel hygroscopic solution [10] initially used as an adjunctive rinse of tooth root canal systems to enhance the removal of post-instrumentation smear-layer and also as an adjunct to subgingival instrumentation in periodontitis [22] or in peri-implantitis treatment [11] due to its proven effectiveness against bacterial dental biofilms [10,41,42,43]. Unlike other commercial products, HI is an “active” cleanser that detaches and effectively removes infectious materials and biofilm from the periodontal pockets. HI simply desiccates certain kinds of oral tissue surfaces to the point of denaturation, but it does not have sufficient desiccation intensity to cause destructive reactions nor does acidify tissue [36]. Associated pre- and post-subgingival instrumentation with HI irrigations reduce the amount of mechanical scaling of subgingival root surfaces that needs to be delivered in order to obtain good clinical outcome [36]. Regarded from a COVID-pandemic perspective, HI reduces the subgingival instrumentation-working time and the eventual SARS-CoV-2 load in the airborne particles induced through subgingival instrumentation [44]. The above-mentioned properties, in addition to the reduced cytotoxicity of HI in comparison with PS and PF, recommend it as a useful adjunctive tool in subgingival instrumentation approaches.

The reports on sodium hypochlorite cytotoxicity seem somehow contradictory. Sodium hypochlorite—the active substance in the PS—exerts a negative impact on non-human MSCs [27,28] or on periodontal ligament fibroblasts [45]. Additionally, SEM analyses reported an inhibitory effect of 0.5 mg/mL sodium hypochlorite on cell adhesion [28]. Other studies have found that there is no influence on cell survival and spread on PS treated-dentin discs when PS is thoroughly rinsed [46]. No data is available on its influence on gMSCs.

As our study observed, several studies also reported a round or thread-like shape of cells after contact with PS, associated with features of cell suffering [28]. PS acts by disrupting bacterial biofilms as wells as by dissolving degenerated tissues through the chemical reaction of sodium hypochlorite with the contained amino-acids to form N-monochloroamino acids, which minimize the detrimental effects of the hypochlorite on healthy tissues [47].

Although there are no direct reports on the effect of PF on oral MSCs, some studies show that silver nanoparticles of 15–20 nm at concentrations of 10 to 1,000 μM result in a time- and concentration-dependent cytotoxic effect on human periodontal ligament cells [32]. In contrast, other studies report that treatment with up to 100 μM silver nanoparticles do not impact human periodontal ligament fibroblast viability [48]. Some potential biohazards related to the toxicity of silver nanoparticles consequent to oral exposure from mouthwash, accidental ingestion and professional exposure to aerosols have been mentioned [31].

The three products underwent additional cytotoxicity tests to the standard ones because responses to dental materials differ among cell types [49]. Thus, due to local products’ proximity with gingival tissues, it seemed important to study their biocompatibility on gingival-derived cells [50]. Moreover, because the periodontal progenitor cellular pool plays an important role in local healing after subgingival application of local antimicrobials, the present study evaluated the influence of commercial products on gMSCs.

Diluting phenomena of the adjunctive subgingivally used products in the periodontal pockets could hardly be previewed. The lack of cytotoxicity appreciation from this perspective could be considered a limitation of our study. The continuous secretion of the gingival crevicular fluid eliminates the antimicrobials from the subgingival areas [51], which renders living tissues less susceptible to the toxic effects of drugs than cultured cells [45]. The deleterious effect of the commercial products, and mostly of PF on gMSCs, warrants caution when used in clinical practice. Nonetheless, our results may not correspond to the in vivo reality and thus the information provided by our study should be considered judiciously.

As previously described and consistent with prior analyses [39,52], our study observed that after mechanical instrumentation with Gracey curettes, the samples were coated with a smear layer, rendering smooth homogenous surfaces. Generally, curette instrumentation is known to form a thin smear layer [52]. Notwithstanding, other reports show that hand curettes produced remarkable surface alterations modifying the root morphology and roughness [53] and exhibiting a thick smear layer. However, the root surface roughness does not impede the success of mechanical subgingival instrumentation [54], although it would significantly increase soft and hard deposit accumulations. Therefore, the exact role of root surface roughness is still not completely agreed [55].

We observed that samples treated with HI show areas of continuous smooth compact smear layer alternating with areas of partially opened dentin tubuli. Other SEM examinations reported that HI efficiently removed the smear layer in the root canal system [56], but no information on its effect on root surfaces after subgingival treatment is available. PS-treated samples demonstrated relative smooth surfaces at low and high magnifications, which is in agreement with other reports [46]. However, partially opened dentin tubuli were also present. Samples treated with PF were rough and displayed abundant deposits. It seems that both HI and PS only partially removed the smear layer, while PF was even less efficient in removing instrumentation debris. It is possible that the smear layer could partially be dislocated by PF but not removed by rinsing. Smear layer persistence could induce clinical problems related to connective tissue re-attachment stability.

The use of root fragments to observe the effect of adjunctive antimicrobial products and mechanical instrumentation on the attachment of MSCs closely recreated a daily-practice model and is considered to be a strong point of the present study, that – unlike other reports—did not use enamel bovine fragments [46] or human dentin [57]. However, the majority of our samples displayed partially opened dentin tubuli, which signifies that the cementum elimination was performed by mechanical treatment although a minimal curette instrumentation of the roots was done.

Additional in vitro testing is required to further characterize the influence of adjuvant antimicrobial products on different types of periodontal cells and to observe the experimental variables that may increase cell adhesions on roots.

## 5. Conclusions

The three commercial products used as subgingival adjunctive antimicrobials to mechanical instrumentation induced a dose-dependent cytotoxicity in terms of reduced proliferation and viability of gMSCs, as well as modifications of cell shapes on SEM images.

The blend of sulphonic/sulphuric acids seems to have been better tolerated than sodium hypochlorite and the silver nanoparticle-based product, but these results are difficult to translate in clinical practice.

Repopulation of root surfaces was possible following SRP associated with antimicrobial product applications, although a scarce distribution of gMSCs was observed. HY and PS provide more stable root surfaces due to a more efficient removal of the smear layer. However, possible side effects of these antimicrobials, such as the opening of dentin tubuli and consecutive dentin hypersensitivity, should be considered from a clinical point of view.

## Figures and Tables

**Table 1:** Characteristics of the antiseptic products.

Product	Manufacturer	Composition	Indication
HYBENX^®^ [35,36] (HI)	Epien Medical, St Paul, MN, USA	*Hygroscopic solution* Sulfonated phenolics (60%) Sulfuric acid (28%) Water (12%)	Oral Tissue Decontaminant Removal of plaque biofilm Topical irrigation during scaling and root planing Topical rinse/solution for oral cavity Adjunctive rinse of tooth root canal systems in endodontic treatments
Perisolv^®^ [29] (PS)	Regedent AG, Zurich, Switzerland	*Gel*: Amino acids (glutamic acid, leucine, lysine) Sodium chloride, Sodium carboxymethyl cellulose Ultrapure water *Liquid*: Sodium hypochlorite solution 0.95%	Adjunctive therapy to scaling and root planing in periodontitis and peri-implantitis due to inhibitory bacterial effects and biofilm elimination
Perioflush^®^ [37] (PF)	Dental Life Sciences LTD, Wigan, UK	*Water based solution*: Ultrapurified water Silver nanocolloid solution Sodium nitrate Apple, mint flavours Lactic acid Phosphoric acid	Rinsing and cleaning the gums and oral mucosa Rinsing around bridge spans, erupting teeth, around implant areas, overhanging fillings, interdental spaces, after scaling

## Data Availability

The datasets generated during the current study are available from the corresponding author on reasonable request.

## Supplementary Materials

The following are available online at https://www.mdpi.com/article/10.3390/ma14175049/s1, Figure S1: Steps in root sectioning. Silicon mounting template containing resin-embedded teeth (a). Fragments of longitudinally sectioned roots (b); Root samples (c). Table S1: CCK8 and MTT test values.
