# Supplementary material for: Cytotoxic Effects on Gingival Mesenchymal Stromal Cells and Root Surface Modifications Induced by Some Local Antimicrobial Products Used in Periodontitis Treatment
Source: Materials (Basel). 2021 Sep 3;14(17):5049. doi: 10.3390/ma14175049 (PMC8434495; doi:10.3390/ma14175049)
Supplement: Supplementary file 1 [file materials-14-05049-s001.zip › materials-1330264-supplementary.pdf]

materials

MDPI

Supplementary Materials

Cytotoxic Effects on Gingival Mesenchymal Stromal Cells and Root Surface Modifications Induced by Some Local Antimicrobial Products Used in Periodontitis Treatment

Irina Lupșe [1,†], Emoke Pall [2,†], Lucian Barbu Tudoran [3,4], Adriana Elena Bulboacă [5], Andreea Ciurea [6], Iulia Cristina Micu [6], Alexandra Roman [6,*], Ada Gabriela Delean [7], Alexandrina Muntean [1] and Andrada Soancă [6]

[1] Department of Paediatric Dentistry, Faculty of Dental Medicine, Iuliu Hațieganu University of Medicine and Pharmacy, Avram Iancu St., No. 31, 400083 Cluj-Napoca, Romania; irinalupse@yahoo.com (I.L.); ortoanda@yahoo.com (A.M.)

[2] Department of Infectious Disease, Faculty of Veterinary Medicine, University of Agricultural Sciences and Veterinary Medicine, 3-5 Mănăștur St., 400372 Cluj-Napoca, Romania; pallemoke@gmail.com

[3] Department of Molecular Biology and Biotechnologies, Faculty of Biology and Geology, Babeş-Bolyai University, Clinicilor St., No. 5-7, 400006 Cluj-Napoca, Romania; lucianbarbu@yahoo.com

[4] Electron Microscopy Integrated Laboratory (LIME), National Institute for Research and Development of Isotopic and Molecular Technologies, INCDTIM, 67-103 Donath St., 400293 Cluj-Napoca, Romania

[5] Department of Pathophysiology, Iuliu Hațieganu University of Medicine and Pharmacy, 4-6 Victor Babeș St., 400012 Cluj-Napoca, Romania; adriana_bulboaca@yahoo.com

[6] Department of Periodontology, Faculty of Dental Medicine, Iuliu Haţieganu University of Medicine and Pharmacy, Victor Babeş St., No. 15, 400012 Cluj-Napoca, Romania; andreea_candea@yahoo.com (A.C.); i.cristina.micu@gmail.com (I.C.M.); andrapopovici@gmail.com (A.S.)

[7] Department of Odontology and Endodontics, Faculty of Dental Medicine, Iuliu Haţieganu University of Medicine and Pharmacy, 400012 Cluj-Napoca, Romania; adadelean@yahoo.com

* Correspondence: veve_alexandra@yahoo.com

† The authors contributed equally to the present study and can be regarded, therefore, as being main authors.

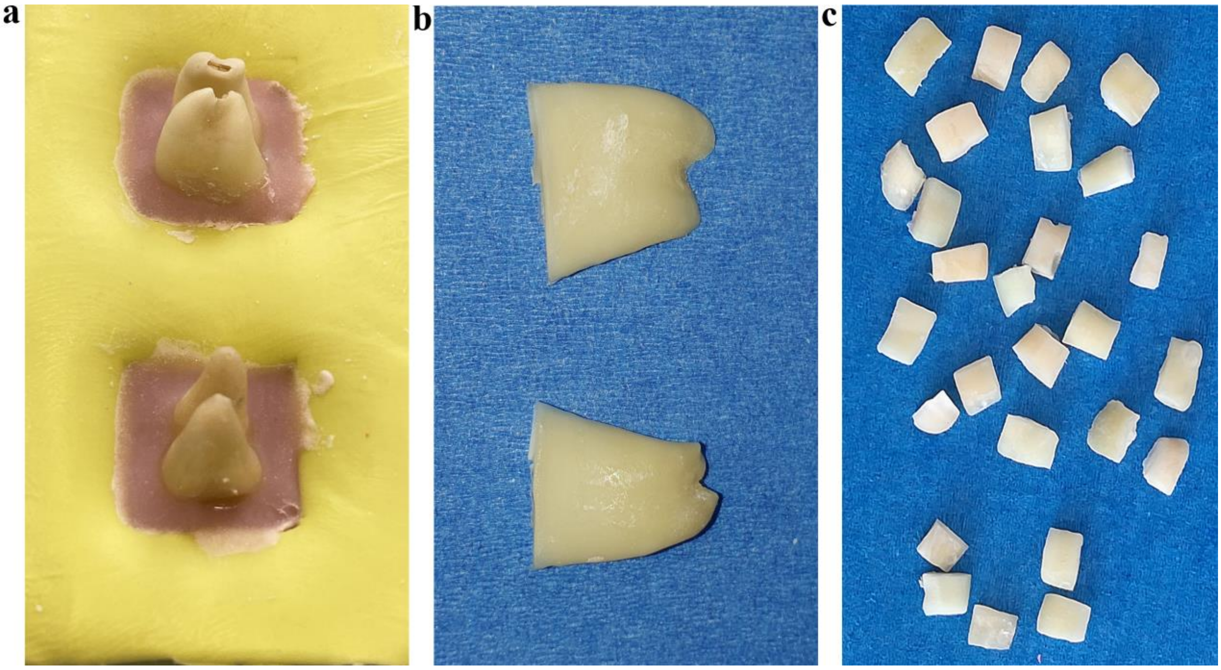

Figure S1. Steps in root sectioning. Silicon mounting template containing resin-embedded teeth (**a**). Fragments of longitudinally sectioned roots (**b**); Root samples (**c**).

Table S1. CCK8 and MTT test values.

Test	Time	Product	Concentration (Mean ± SD) (Optical Density for CCK, % Cell Viability for MTT)					
			**50%**	**20%**	**10%**	**5%**	**2%**	**Control**
**CCK test**	**48 h**	HI	0.24766 (0.01234)	0.262 (0.03005)	0.26266 (0.00838)	0.278 (0.006)	0.275 (0.00458)	0.285 (0.02893)
		PS	0.177 (0.00793)	0.17433 (0.015144)	0.25966 (0.00321)	0.27266 (0.00665)	0.27833 (0.00702)	0.285 (0.02893)
		PF	0.14766 (0.01422)	0.18033 (0.00763)	0.20666 (0.00929)	0.207 (0.05494)	0.21266 (0.04099)	0.285 (0.02893)
	5 days	HI	0.47933 (0.02411)	0.51 (0.01081)	0.51533 (0.00513)	0.546 (0.007)	0.53833 (0.01975)	0.55266 (0.01059)
		PS	0.36366 (0.00737)	0.418 (0.07015)	0.52066 (0.0674)1	0.53366 (0.04416)	0.54133 (0.01205)	0.55266 (0.01059)
		PF	0.41733 (0.02893)	0.43066 (0.04373)	0.42433 (0.0155)	0.45633 (0.04801)	0.494 (0.01928)	0.55266 (0.01059)
**MTT test**	24 h	HI	60.50815 (6.03281)	75.42372 (10.23595)	82.62711 (14.1601)	81.77966 (8.98677)	80.9322 (2.49066)	100 (0)
		PS	63.90374 (10.70935)	71.39037 (10.1317)	92.51336 (17.4227)	96.39037 (14.0836)	95.18716 (3.06329)	100 (0)
		PF	57.35294 (6.03281)	70.72192 (6.03281)	70.18716 (14.1601)	75 (8.98677)	78.877 (2.49066)	100 (0)

HI=HybenX; PS=Perisolv; PF=Perioflush; CTRL=Control.
